# Annotations

**Published:** 1899-07-08

**Authors:** 


### Annotations.

### A Coroner's Inquest Without a Jury.

By an amendment to the Code of Criminal Procedure of New York State, which is to take effect on September 1st, coroners will be empowered to conduct inquests without the aid of a jury, except in counties which contain cities of the first-class. However, as is pointed out by the New York *Medical News*, although the new rule does not touch the City and County of New York, it is already legal there for the coroner to subpœna a properly qualified physician, who shall view the body or make an autopsy as required, and for the testimony of such physician, and that of any other witnesses that the coroner may find necessary, to constitute an inquest, juries not being called in. In England we are in the curious position that while the coroner and his officer can collect informal evidence, and on the strength of it decide whether or not to hold an inquest, the coroner does not appear to be able to obtain sworn evidence except in the presence of a full jury. Hence on the one hand many useless inquests, and on the other many cases of people dying suddenly and yet having no inquest held upon them because of the village gossips holding that they had suffered from "heart disease," or, in the case of children, that they had died from "convulsions." It is but a small alteration of the law which is wanted to render the office of coroner one of real utility to the community; but the reform which is required is one which is thought to infringe on the rights of that ancient and, so far as the coroner's court is concerned, we fear, somewhat beery institution the British jury, so that in all likelihood things will go on as they are for a good many years to come, notwithstanding the example being set us in the United States.

### Collective Research.

Under the influence, perhaps, of that sort of despair which is apt to overwhelm the scientist when he considers the vastness of the field from which knowledge of Nature's laws has yet to be sought, a tendency again and again crops up to ask for collective rather than individual research, and no one could have put the case for the collective method better than it was put by Mr. Haffkine at the dinner of the Epidemiological Society. He showed not only how enormous is the field awaiting investigation, but how varied are the kinds of knowledge which must be brought to bear upon each problem if results of real utility are to be obtained. The latter, we fancy, will be found to give the key to the position which will be taken by collective investigation in the future. The phrase "collective investigation" has been too much associated in the past with efforts, by sheer multitude of observers, to "clear the ground," as the saying is, in regard to certain "facts." In future we shall probably find that, instead of collecting facts, we shall try to look at each fact from many sides. It is here that the necessity for co-operation and the helplessness of the observer who works single-handed become most apparent. As Mr. Haffkine said, "not only in such a vast complex of phenomena as that represented by an epidemic, but upon one square yard of garden soil, every one of them would be able to point out quite a definite set of associated or dissociated objects of investigation, such as might keep occupied all the institutions of the world for physical, chemical, mineralogical, zoological, botanical, even sociological, and many other researches during . . . a century to come." It is not by a mere multitude of bayonets, a mere repetition of the same arm and the same method over and over again, as in the older forms of "collective investigation," that the secrets of nature are to be attacked, but by every arm of the service, by horse, foot, and artillery, by the chemist, the biologist, and the epidemiologist pouring a concentrated fire upon the subject of inquiry. This is organised specialism, and needless to say the general, the organiser, is as necessary as the private. "It is only by introducing a whole service of students into a co-ordinate machinery of inquiry, by transforming a scientific school into one collective investigator, animated with trust in their teacher and guided by one idea, that . . . useful truth can be gathered, and that the study of calamitous phenomena, such as are represented by epidemics, can yield effective information for the benefit of a country and of mankind as a whole." Such, then, is the ideal of collective research.

### Club Practice and Expensive Drugs.

Some time ago we pointed out the very serious position in which club surgeons, and other medical men who accept contracts for the supply of "attendance and medicine" at very low rates, might find themselves in consequence of the high price of antitoxin. Clearly a contract doctor must either deny the efficacy of such a drug, or he must supply it for suitable cases, even at a ruinous loss to himself, or, finally, he must openly acknowledge what we think is sufficiently obvious, that his halfpenny a week, or whatever it is, does not cover, and was never intended to cover, the supply of the best drugs. But medical men have always scouted the little innuendos that have so often been thrown out concerning the true inwardness of club practice, notwithstanding the facetious details given by Albert Smith, Dickens, and other more or less veracious historians. In fact, as good, honest members of a noble profession, what else could we do? Still, a few of us have had our doubts. Indeed, here and there an honest and ingenuous person has admitted that in arranging for his dispensing to be done for him by a neighbouring chemist he has made "special terms" for club patients, and now a still more ingenuous person writes to the *British Medical Journal* in great trouble because, having recommended a certain expensive proprietary medicine to a club patient, on condition that the patient should pay for it, the "lodge" immediately demanded that the doctor should supply the medicine at his own cost, he having contracted to supply "proper and sufficient medicine." This is a case exactly analogous to that of the antitoxin. The medical man evidently thought that the drug was an appropriate one to give, yet he "could not himself afford to supply it under the club contract." Why, then, did he accept the contract at such a rate? And why do men so often accept contracts at rates which they may be certain will not allow them to have a free hand, or to give what they consider the best for their patients? We have always thought that the friendly societies and the medical aid associations and other contracting bodies have been greatly to blame in grinding down their medical officers as they have done in regard to price, but it would be childish to pretend that the blame is all on one side, and that none attaches to the medical men themselves, who accept contracts at rates which cannot possibly enable them to act honestly by their patients. The General Medical Council has lately given its sanction to the creation of a Board of Conciliation between the friendly societies and their medical officers. But before any good can be done, or any so-called conciliation can be entered upon, it must be recognised and admitted that the fees offered by many of the societies are not sufficient to be remunerative. Let us begin by clearing away a lot of sham. The doctors know, the secretaries of the clubs know, and even the patients themselves must know that the payment which is often given is not remunerative, and that although it may pay for sympathy and soft sawder, it cannot pay for scientific treatment.

